# Supplementary material for: Patterns of Intron Gain and Loss in Fungi
Source: PLoS Biol. 2004 Nov 30;2(12):e422. doi: 10.1371/journal.pbio.0020422 (PMC532390; doi:10.1371/journal.pbio.0020422)
Supplement: Table S1 — Also available at http://genes.mit.edu/NielsenEtAl/. (4.3 MB ZIP). [file pbio.0020422.st001.zip › NielsenEtAl/html/548.html]

AN6726.1.NCU06764.1.MG07165.2.FG04410.1


```
 CLUSTAL W (1.82) Multiple Sequence Alignments - Introns Inserted


Sequence 1: NCU06764.1	274 aa
Sequence 2: MG07165.2	266 aa
Sequence 3: AN6726.1	278 aa
Sequence 4: FG04410.1	279 aa
Alignment Length: 286 aa
Number Identitical Residues: 209 aa
Alignment Score (without introns) 8318


MG07165.2 	MADRYSFSLTTFSPS2GKLVQIA1T----------------~-NGIVLATEKKSSSPLTD
NCU06764.1	MADRYSFSLTTFSPS2GKLVQIA1LGI------------KA1TNGIVLATEKKSSSPLAD
FG04410.1 	MADRYSFSLTTFSPS2GKLVQIE1YALNAVNQGITALGIKA1TNGIVIATEKKSSSPLAD
AN6726.1  	MADRYSFSLTTFSPS2GKLVQIE1YALNAVNQGVTALGIKA1TNGIVLATEKKSSSPLID
          	*************** ******   . .: ... :: . .: :****:********** *

MG07165.2 	PSSLSKISLVTPNIGMVYAGMGPDYRVLVDKARKVSHSGYKRIYNEYPPTRILVQDVARV
NCU06764.1	PSSLSKISLITPNIGMVYSGMGPDYRVLVDRARKVSHTGYKRIYNEYPPTRILVQDVARV
FG04410.1 	QSSLSKISDITPNIGMVYSGMGPDYRVLVDRARKVSHTEYKRIYNEYPPTRILVQDVARV
AN6726.1  	PPSLSKISLITPDIGMVYAGMSPDYRVLVDKARKVSHTGYKRIYNEYPPTRILVQDVARV
          	 .****** :**:*****:**.********:******: *********************

MG07165.2 	VQEATQSGGVRPYGVSLLIAGWDEGIPPED---DESAGDRMIEDEDGEKKKISGKTGGIL
NCU06764.1	MQEATQSGGVRPYGVSLLIAGWDEGILPEDELEEQKSKDEMVADDGETKKKATGKTGGIL
FG04410.1 	MQEATQSAGVRPYGVSLLIAGWDEGIEPED-------EEDDKSQEDSEEKKVNRKTGGIH
AN6726.1  	VQEATQSGGVRPYGVSLLIAGWDEGVEPET--------AQAQKGEEEEPKKATGKTGGIL
          	:******.*****************: **               :    ** . ***** 

MG07165.2 	KGGPMLYQVDPSGSYFPWKATAIGKSAASAKTFLEKRYTEGLELEDAVHIALLTLKETIE
NCU06764.1	KGGPMLYQVDPSGSYFPWKATAIGKNATTAKTFLEKRYTEGLELEDAVHIALLTLKETIE
FG04410.1 	KGGPMLYQVDPSGSYYPWKATAIGKSATKAKTFLEKRYSEELELEDAIHIALLTLKDNIE
AN6726.1  	KGGPSLYQVDPSGSYYPWKATAIGKHATSAKTFLEKRYTEGLELEDAIHIALLTLKETIE
          	**** **********:********* *:.*********:* ******:********:.**

MG07165.2 	GEMNGDSIEIG1IVGPPADHLLGVEGVEGAQGPRFRKLSPQEIEDYLTNL
NCU06764.1	GEMNGDTIEIG1IVGPPADHLLGVEGVEGAKGPRFRKLSPQEIEDYLTNL
FG04410.1 	GEMNGDSIEIG1IVGAPADHLLGLEGVEGATGPRFRKLTPQEIEDYLTSL
AN6726.1  	GEMNGDTIEIG1IVGPPADHLLGYEGVEGARGPRFRKLTKEEIEDYLTSL
          	******:**** ***.******* ****** *******: :*******.*
```
